# Therapists’ experiences with implementing new documentation practices for low back pain in electronic health care records: an interview study

**DOI:** 10.1186/s13104-023-06567-w

**Published:** 2023-10-26

**Authors:** Anne Katrine Skjølstrup Toftdahl, Stine Ibsen, Louise Bilenberg Pape-Haugaard, Allan Riis

**Affiliations:** 1 Department of Physiotherapy, University College of Northern Denmark, Aalborg East, Denmark; 2 Department of Health Science and Technology, Aalborg University, Aalborg East, Denmark; 3 Research Unit for General Practice in Aalborg, Aalborg University, Aalborg East, Denmark

**Keywords:** Electronic health care records, low back pain, data quality, Physiotherapy, occupational therapy, Collect once use many times

## Abstract

**Objective:**

Clinical practice is constantly changing with new guidelines being published, changes in patients’ preferences but also by new qualitative requirements for therapists and institutional surveys on delivered care. Electronic health records (EHR) are used for all these purposes. We involved physiotherapists and occupational therapists in an intervention to change documentation practice in their electronic health record for low back pain (LBP) and later evaluated the feasibility of the new health records. The aim of the present study was to explore therapists’ experiences working with the new EHR.

**Results:**

Three themes were identified thru interviews: (I) Facilitators and motivators towards implementation, (II) Changing routines as a group and (III) Obstacles against successful implementation. This study identifies a need for involving therapists and management for successful change of electronic health care records usage in municipalities. However, difficulties were encountered in meeting documentation of practice requirements and obtaining sufficient data quality in the EHR for data to be used for daily use, quality assessment and research. In this small descriptive study, developing an EHR that simultaneously serves treatment plans, quality assessment, and research purposes was not expressed being feasible. Further research in this area is needed.

**Supplementary Information:**

The online version contains supplementary material available at 10.1186/s13104-023-06567-w.

## Introduction

Low back pain (LBP) causes more global disability than any other condition [1], and the quality of data on LBP in the municipal Electronic Healthcare Records (EHR) has been found to be insufficient in regards to documenting changes in pain and physical function [2].

Clinical practice is constantly changing with new guidelines being published, changes in patients’ preferences but also because new qualitative requirements are introduced for health care professionals [3]. The therapeutic EHR is used to document practice and support the therapist in delivering the planned treatment and follow-up on treatment effects. Under ideal circumstances, an evidence-based practice (EBP) is mirrored in the EHR [4]. If this is done consistently using validated and uniform methods, data from EBP may be used for several purposes; e.g. to support decisions, conduct research, monitor quality, produce management reports and support reimbursement [4]. This has been described as the principle of ‘Collect Once - Use Many Times’ (COUMT) [5], a principle that is increasingly being used in medical health care [6]. However, existing literature highlights challenges concerning the quality of the reported data in the EHR, such as imprecise and inadequate use of outcome measures, inconsistent registration and the use of outcome measures that have not been formally validated or otherwise tested for measurement abilities [2, 7]. Together with the management at Aalborg Municipality and municipal physiotherapists and occupational therapists, we facilitated the development of an intervention to change documentation practice. The aim of the present study was to explore therapists’ experiences working with the new EHR.

## Main text

### Description of the intervention

The therapist team was educated in the recommendations regarding relevant clinical data for patients with chronic pain as recommended by the the Initiative on Methods, Measurement, and Pain Assessment in Clinical Trials (IMMPACT) guidelines [8]. AKST presented a selection of standardised tests to the therapist team: A numerical rating scale (NRS) [9], the Danish version of the EQ-5D-5 L [10], Tampa scale for fear of movement [11], Timed Up and Go [12], Straight Leg Raise [13], Chair Stand Test [14], Roland Morris Disability Questionnaire [15], Patient-Specific Functional scale [16], Patients’ Global Impression of Changes, Oxford Scale, Oswestry Disability Index [17], Örebro Musculoskeletal Pain Questionnaire [18], a neurological screening and the Neck Disability Index [19]. In addition, therapists participated in group sessions to discuss how data quality could be improved. The team agreed on including specific goals for each patient and on a standard structure including a NRS, [9], Patient-Specific Functional scale [16], and EQ-5D-5 L [10] at baseline and at end of treatment. The Template for Intervention Description and Replication (TIDieR) statement is used to describe the intervention [20] (Supplemientary material).

## Methods

Interviews were used to study therapists’ experiences with the changes in documentation practice and their way of being-in-the-world (their clinical practice), a hermeneutic approach was applied. Kotters’ framework was used in a barrier analysis [21]. Reporting is performed in accordance with the COREQ (consolidated criteria for reporting qualitative research) checklist [22]. The interviews were conducted by AKST. She is senior lecturer at a university college in the North Denmark Region. She is a physiotherapist with a master’s degree in clinical science and technology. She had interview expertise and had received extensive training in posing open-ended questions, active listening and probing as part of her work and during her education. AKST was also responsible for facilitating the change of EHR. Participants participated in the development of the new EHR structure. Thus, the participants knew the interviewer’s credentials and motivations prior to the interviews but had no personal knowledge of AKST.

### Clinical setting

A total of 16 physiotherapists and occupational therapists were involved in developing the newEHR structure. Therapists had to be involved in changing the EHR to be eligible to participate with- one-to-one interviews. We had identified 5 with a strong engagement in the development and implementation of the changes, these five were invited to be interviewed. However, one did not accept to participate. All participants provided written informed consent before being interviewed. All interviews took place at a municipal therapeutic rheumatology unit in the North Denmark Region. The reason for not conducting a focus group interview, was based on the assumed risk of restricting the openness during interviewing, since some participants had been strongly supporting some of the changes while others had been more interested in other changes. Interviewing was conducted by AKST together with the interviewed person; no others were present. AKST used a semi-structured interview guide, which wasbased on the literature and feedback received during its development and pilot tested on a physiotherapist not participating in the study (Table 1). All interviews were audio-recorded by use of an OLYMPUS Digital Voice Recorder (WS-853) and no field notes were taken.

**Table 1:** Interview guide

Research question	Interview question
How do the participants experience benefits and challenges during the changing of their documentation practice? (workshops, meetings, teaching and working in teams)	How have you experienced the implementation process worked in practice? Did you learn anything during the process? Give three examples of how this implementation has affected your daily practice? Do you experience that, you learned something from this process - and what? How could the three workshops have been improved to facilitate the implementation better? - Why? What was most beneficial in the workshops in which you participated? - Why? How do you experience your development concerning documentation? How do you experience this? How do you think the team’s development has been concerning documentation? How do you experience this? Do you have any other feedback about the way this implementation process progressed? Me? Management? Aalborg Municipality as an organisation?
How do the participants experience benefits and challenges regarding their daily work after the changes in documentation practices?	How do you experience the implementation process in which you and your team have participated? Do you find it meaningful to participate in this project? Are you motivated to change data quality in your documentation (EHR)? How do you experience your development has been with documentation? How do you experience this? How do you feel the team’s development has been concerning documentation? How do you experience this? What challenges do you experience daily related to complying with the agreements you have made in the team? (e.g. professional, practical, logistical, the theme work, the management, technical, Other?) What do you experience that works well with the new documentation practice? What challenges you? (e.g. professional, practical, logistical, the theme work, the management, technical, Other?) What do you need in the future to stick to the new practice? (e.g. knowledge, practicalities, logistics) What do you do daily to maintain the implementation? What do you do, in your team, to keep each other in the implementation process? Do you have other ideas for how the implementation process can continue to be maintained and/or improved?

### Data analysis

Data were coded manually by AKTS and the coding tree was therefore described by AKTS. The anonymised text files were coded using NVivo 12. The coded material was analysed by thematic text analysis as described by Braun and Clarke in seven steps [23]. The analysis was performed by AKST and validated by an experienced researcher and physiotherapist, who was not authoring the present paper.

## Results

Five possible participants were invited for interviews, however, one decided not to participate. In November 2018, two women and two men with a range in experience between 3 and 25 years participated took part in 31–49 minutes one-to-one interviews. Three themes emerged I) Facilitators and motivators of implementation, II) Changing routines as a group and III) Obstacles hampering successful implementation. When adding requirements for the EHR to be used for research and quality monitoring, it can be difficult to decide upon the best structure. Balancing multiple purposes in the EHR to achieve meaningful change without demanding excessive resource consumption was also found to be challenging. As the therapist navigates in a complex clinical ‘real-life’ setting, whereas standardised structures simplify the complexity, this imbalance created an obstacle for changing the documentational practice with a focus on COUMT. Together this seriously challenges the use of COUMT. The themes are elaborated below.

### Facilitators and motivators of implementation

The participants expressed that the external facilitation increased their motivation.

*’That someone facilitates the process from the outside increases the energy and interest level in the group, and some of the questions initiated interesting discussions among us’* (Participant).

External facilitation of the implementation and learning process was found necessary to attain documentation changes and ensure data quality. However, it was also expressed that improving data quality in a constantly changing clinical setting with changing guidelines and local treatment procedures requires frequent adaptations of the EHR structure.

*’We have actually asked for this, that someone from the outside could come at tell us about the newest evidence as we wish to do our best – but we don’t have the time to stay updated ourselves’* (Participant).

Awareness of the need for continuously working with the structure of EHR and involving external facilitation could avoid that therapists otherwise return to their old documentation habits.

‘I*t has become very clear, the fact that we work and learn new things in different ways’* (Participant).

External and internal motivation is important when changing routines. The therapists described how external facilitation reinforced the external motivation system by keeping focus and momentum during the implementation. However, the therapists described how maintaining their internal motivation was challenging in daily clinical practice when changing working procedures. Here, the extrinsic motivation must be translated into intrinsic motivation, which was expressed to facilitate retention of change in the organization.

*‘It is easier for newly educated staff to work with this and get used to the mindset – but also seeing the meaning and purpose of this’* (Participant).

The therapists described how old habits or experiences along with differences in routines among the therapist challenged this internal motivation. However, therapists expressed that working together as a group towards a common goal increased their motivation.

### Changing routines as a group

In a team of skilled therapists with a high degree of professionalism and a high level of individual decision-making competency, respecting the therapists’ individual professionalism was expressed to be important. These individual initiatives and professionalism must be balanced, respected, recognised and considered throughout the implementation process.

‘*I would have had a hard time - in terms of implementing it, if I had to change too much, I think it would be too extensive work’* (Participant).

The therapists described how changing habits and making new routines takes time and requires resources. When the necessary resources are unavailable, it is experienced as difficult to maintain new routines. Therefore, it is important to acknowledge time requirements when implementing change in work routines.

‘*I am used to being a practitioner and then I’m blown away when I am expected to sit and reflect on my own practise and why I do what I do’* (Participant).

Involvement and ownership of the intervention to change were expressed as essential to increase participants’ compliance. For example, the participants did not share definitions of high data quality and they had limited common understanding of the needed contents in the documentation structure. Having a common language about the documentation structure was experienced to facilitate change adherence. Also, changing clinical practice requires time to reflect and thereby to allow for new learning to be implemented.

*‘it has been good for us to discuss this physio- and occupational therapists together - some is fact, and some is evidence, and we have to relate to this so. But the other profession has a slightly different approach than we have’* (Participant).

Learning and reflection on new routines were considered important to create a sense of relevance of these changes; and without a feeling of relevance, the new routines were believed to be poorly implemented in daily clinical practice.

*‘I certainly think we have learned something both from thinking about the method and why is it precisely I do things the way I do? What is my purpose? It has been good for me to return to. Also, to be exact about what I write. I think about it a lot, and it’s my feeling that my colleagues do the same’* (Participant).

#### Obstacles hampering successful implementation

The therapists expressed some obstacles toward successful implementation. Obstacles such as lack of time, different organisation and cultures within each team individual working methods among therapists, lack of motivation to implement change, and difficulties in agreeing on a uniform structure were expressed as barriers.

‘*And now we implement it, and it is a project on its own. This is because for many years we have done it in our own way’* (Participant).

### Limitations

The participants had all been involved in developing the intervention workshops and had extensive experience with the new EHR structure this strengthens the power of information; however, findings may be different for therapists who have not been involved in developing the changes and therapists not being involved in the decisions to change practice might have more barriers to use the new EHR structure. Also a study sample of four should be interpreted with great caution.

## Conclusion

In this small descriptive study, developing an EHR that simultaneously serves treatment plans, quality assessment, and research purposes was not expressed being feasible. Further research in this area is needed.

### Electronic supplementary material

Below is the link to the electronic supplementary material.

Supplementary Material 1

## Data Availability

The data that support the findings of this study are available from the corresponding author [AKST], upon reasonable request.

## Abbraviations

COUMT: Collect Once - Use Many Times
EHR: Electronic Health Record
IMMPACT: Initiative on Methods, Measurement, and Pain Assessment in Clinical Trials guidelines
LBP: Low Back Pain
