# Supplementary material for: Therapists’ experiences with implementing new documentation practices for low back pain in electronic health care records: an interview study
Source: BMC Res Notes. 2023 Oct 26;16:293. doi: 10.1186/s13104-023-06567-w (PMC10605498; doi:10.1186/s13104-023-06567-w)
Supplement: Supplementary file 1 — Supplementary Material 1 [file 13104_2023_6567_MOESM1_ESM.docx]

Supplementary material

Appendix 1. TIDieR template

|  | BRIEF NAME |  |
| --- | --- | --- |
| 1. | Provide the name or a phrase that describes the intervention. | Change of documentation practice for low back pain |
|  | WHY |  |
| 2. | Describe any rationale, theory, or goal of the elements essential to the intervention. | The goal was to develop a documentation practice allowing data to be used to support and document treatment for the individual citizen as well as allowing data to be used for quality development and research purposes. Previous studies have found that involving healthcare professionals in developing the intervention is effective to succeed with implementation of clinical guidelines. |
|  | WHAT |  |
| 3. | Materials: Describe any physical or informational materials used in the intervention, including those provided to participants or used in intervention delivery or in training of intervention providers. Provide information on where the materials can be accessed (e.g., online appendix, URL). | By use of workshops and participatory design we developed a new documentation practice.  Before implementation the EHRs were organised as a well-structured first visit; followed by free prose text structured by the individual therapist. However, the content was defined by the consulting therapist.  After implementation the same structure was used at first and last visit, and the content of these visits was partially pre-defined (including the standardised test). Also, the therapist agreed on “good examples of EHRs’, especially focusing on content, language and clinical goals. |
| 4. | Procedures: Describe each of the procedures, activities, and/or processes used in the intervention, including any enabling or support activities. | Workshop 1)  The purpose was to explore potential barriers and resources in the team regarding implementation of the new documentation practice. The therapist team in the municipal unit was encouraged to attend as many workshops as possible. The workshop was initiated by a teaching session that focused on refreshing initial findings of poor data quality in the municipal EHRs as presented by Toftdahl and colleagues in 2016. Subsequently, a barrier analysis was carried out inspired by the recommendations of the Danish board of health in 2014. A focus group interview was conducted (n=4) to validate and explore findings from the barrier analysis and findings were integrated in the second workshop.  Workshop 2)  The purposes were   1. to educate the therapist in the COUMT- regime and its purposes and hereby increasing the teams understanding for the relevance of high data quality in EHRs and II) illustrating how the evidence-based practice could be transparent in the EHR. After this learning activity, AKTS conducted a focus group interview (n=11) to evaluate and validate findings of the workshop 2. II) to uncover further learning potentials. This evaluation gave input to blind spots of the documentation process, which should be addressed in the following workshop.   Workshop 3)  The purpose was to set the form and content for future data collection in the EHR. The therapy team was gathered and offered the opportunity to participate and contribute. The team started the process by analysing current EHRs to uncover points of interest and causes for variation in documentation. The team decided on two points of attention when choosing the course for future documentation practice. The evaluation gave input to issues needing attention, namely data conformity and data completeness.  Workshop 4)  AKST conducted a series of informal interviews prior to workshop 4 to validate the implementation process so far and to uncover further barriers that needed addressing in this workshop. It addressed both cultural and contextual barriers uncovered in the interviews. |
|  | WHO PROVIDED |  |
| 5. | For each category of intervention provider (e.g., psychologist, nursing assistant), describe their expertise, background and any specific training given. | AKST delivered the intervention. AKST is a female and senior lecture at University College in The North Denmark Region. She is BA Physiotherapist and MSc (Master of Science) in Clinical Science and Technology. She had interview expertise and had received extensive training in interview techniques such as open-ended questions, active listening and probing as part of her work and during her educations. |
|  | HOW |  |
| 6. | Describe the modes of delivery (e.g., face-to-face or by some other mechanism, such as internet or telephone) of the intervention and whether it was provided individually or in a group. | Each workshop was face-to-face meetings with AKST and the therapist team as a group |
|  | WHERE |  |
| 7. | Describe the type(s) of location(s) where the intervention occurred, including any necessary infrastructure or relevant features. | A municipal therapeutic rheumatology unit in The North Denmark Region. The therapeutic team consisted of 12 physiotherapists and four occupational therapists, and they were organized in three units. |
|  | WHEN and HOW MUCH |  |
| 8. | Describe the number of times the intervention was delivered and over what period of time including the number of sessions, their schedule, and their duration, intensity or dose. | Four workshops were held each with at duration of 6-7 hours. At each workshop between 10 and 12 therapists attended aside from AKST. The timeframe of the implementation was approximately one year; from May 2018 (workshop no. one) to May 2019 (workshop no. four). AKST 2-3 days planning each workshop and 2-3 days analysing the previous workshop material. |
|  | TAILORING |  |
| 9. | If the intervention was planned to be personalised, titrated, or adapted, then describe what, why, when, and how. | N/A |
